# A Case of Tricuspid Valve and Papillary Muscle Rupture Due to Entrapment of the Tendon of the Tricuspid Valve by the PENTARAY^TM^ Catheter

**DOI:** 10.1002/ccr3.72069

**Published:** 2026-02-22

**Authors:** Daisuke Yamazaki

**Affiliations:** ^1^ Department of Cardiology Akita Cerebrospinal and Cardiovascular Center Akita Japan

**Keywords:** PENTARAY catheter, percutaneous myocardial ablation, tendon rupture, tricuspid valve

## Abstract

When using multipolar mapping catheters for catheter ablation, take care to pass through the central portion when crossing the valve annulus or chordae tendineae, and avoid applying torque as much as possible.

## 1 Case Presentation

The patient was a 46‐year‐old male who had a premature ventricular contraction (PVC) with palpitation symptoms for the past 5 years. He was taking a beta blocker (carvedilol) for drug therapy and etizolam due to anxiety complications caused by palpitation symptoms. Electrocardiogram revealed PVC at the right ventricular (RV) outflow tract origin. The patient was referred to our hospital and underwent percutaneous myocardial ablation.

**FIGURE 1:**
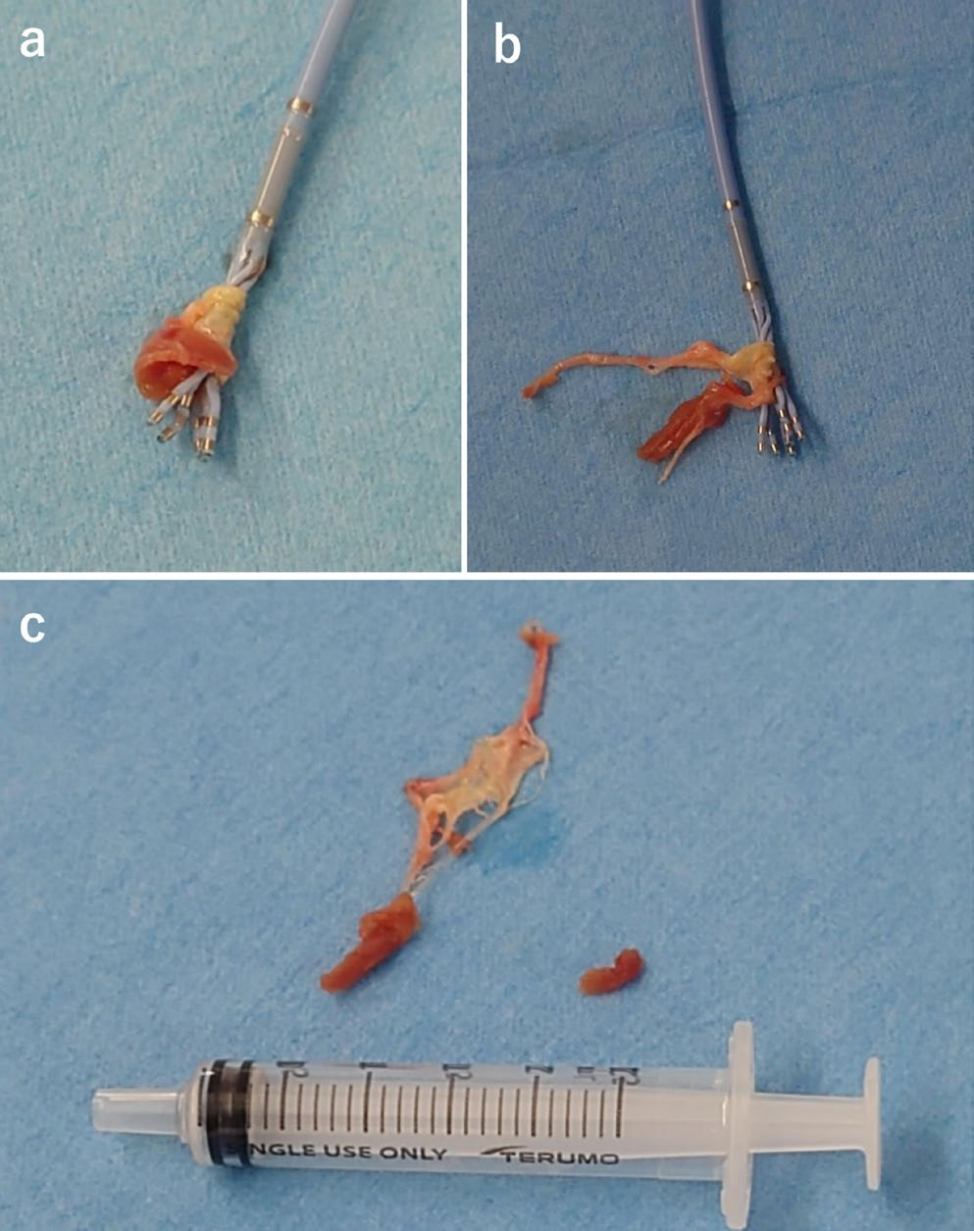
A piece of the tricuspid valve that had become wrapped around the PENTARAY^TM^ catheter. (a) PENTARAY^TM^ catheter was retrieved. At this point, the details of the tissue cannot be determined due to the tight wrapping. (b) Unraveling the wrapped tissue from the legs of the PENTARAY^TM^. Myocardium‐like tissue and cord‐like tissue are visible. (c) The tissue removed from the legs. The papillary muscles, chordae tendineae, and part of the tricuspid valve can be identified.

Mapping of the origin of the PVC with an irrigation catheter (THERMOCOOL SMARTTOUCH^TM^; Biosense Webster, California, USA) suggested an RV outflow tract septum origin. PENTARAY^TM^ catheter (Biosense Webster, California, USA) was used for more detailed mapping of multiple points. The PENTARAY^TM^ catheter was focused on mapping the RV outflow tract septum. The catheter was operated while referencing RAO view's fluoroscopy and CARTO 3D mapping. When the catheter was advanced into the RV outflow tract, there was resistance and passage was difficult. There was also resistance when pulling the catheter. When the catheter was rotated in the same direction to reduce its resistance, the resistance disappeared, allowing the catheter to be removed. Tissue as shown in Figure 1a was wrapped around the leg of the PENTARAY^TM^ and when removed and unfolded, it was a structure that appeared to be part of the papillary muscle and tendon cord/tricuspid valve (TV) (Figure 1b,c). Since the TV was considered injured and dissected, transthoracic echocardiography was performed to confirm the presence of tricuspid regurgitation (TR) and pericardial effusion, which revealed a new moderate degree of TR. Since there was no pericardial effusion and no decrease in blood pressure, catheter ablation was continued. After additional mapping with THERMOCOOL SMARTTOUCH^TM^ again, the RV outflow tract septal wall was cauterized, and the PVC disappeared, completing the procedure. The tissue attached to the PENTARAY^TM^ was submitted for histopathology and confirmed to be the TV, tendon cord and papillary muscle. The patient was discharged from the hospital without developing heart failure. He continues to have outpatient visits; however, he has no symptoms of right heart failure due to TR, and his TR remains mild on echocardiographic evaluation.

## 2 Discussion

Although pericardial effusion and cardiac tamponade are sometimes seen as complications of catheter ablation [1], there are no other case reports of TV rupture. When advancing the PENTARAY^TM^ into the RV outflow tract, the leg of the PENTARAY^TM^ caught on the tendon of the TV. Rotation of the catheter was applied to relieve the resistance of the catheter, which wrapped the tendon cords of the TV around the legs of the catheter and separated the papillary muscle and TV. Based on anatomical location, the ruptured papillary muscle was considered the septal papillary muscle. Rotation is sometimes used to relieve catheter resistance; however, catheters with multiple legs, such as PENTARAY^TM^, tend to wrap around the string‐like structure when rotated. When passing a multipolar mapping catheter through the annulus, it is a preventive measure to reduce the risk of such complications by passing it through the center of the annulus without rotating it. If the multipolar mapping catheter becomes trapped in the chordal area, one direction rotation should be avoided because this may cause the surrounding chordae to become involved.

## 3 Conclusion

During ablation, multipolar mapping catheters are often used due to their ease of mapping; however, caution is required when passing near the tendon chord.

## Author Contributions

**Daisuke Yamazaki:** conceptualization, visualization, writing – original draft, writing – review and editing.

## Funding

The author has nothing to report.

## Ethics Statement

The author has nothing to report.

## Consent

Written informed consent was obtained from the patient to publish this report in accordance with the journal's patient consent policy.

## Conflicts of Interest

The author declares no conflicts of interest.

## Data Availability

The data that support the findings of this study are available on request from the corresponding author. The data are not publicly available due to privacy or ethics restrictions.
